# Belowground neighbor perception in *Arabidopsis thaliana* studied by transcriptome analysis: roots of *Hieracium pilosella* cause biotic stress

**DOI:** 10.3389/fpls.2013.00296

**Published:** 2013-08-13

**Authors:** Christoph Schmid, Sibylle Bauer, Benedikt Müller, Maik Bartelheimer

**Affiliations:** ^1^Faculty of Biology and Preclinical Medicine, Institute of Botany, University of RegensburgRegensburg, Germany; ^2^Faculty of Biology and Preclinical Medicine, Cell Biology and Plant Biochemistry, University of RegensburgRegensburg, Germany

**Keywords:** *Arabidopsis thaliana*, belowground, biotic interaction, *Hieracium pilosella*, interspecific interaction, microarray, pathogenesis-related proteins, root distribution

## Abstract

Root-root interactions are much more sophisticated than previously thought, yet the mechanisms of belowground neighbor perception remain largely obscure. Genome-wide transcriptome analyses allow detailed insight into plant reactions to environmental cues. A root interaction trial was set up to explore both morphological and whole genome transcriptional responses in roots of *Arabidopsis thaliana* in the presence or absence of an inferior competitor, *Hieracium pilosella*. Neighbor perception was indicated by *Arabidopsis* roots predominantly growing away from the neighbor (segregation), while solitary plants placed more roots toward the middle of the pot. Total biomass remained unaffected. Database comparisons in transcriptome analysis revealed considerable similarity between *Arabidopsis* root reactions to neighbors and reactions to pathogens. Detailed analyses of the functional category “biotic stress” using MapMan tools found the sub-category “pathogenesis-related proteins” highly significantly induced. A comparison to a study on intraspecific competition brought forward a core of genes consistently involved in reactions to neighbor roots. We conclude that beyond resource depletion roots perceive neighboring roots or their associated microorganisms by a relatively uniform mechanism that involves the strong induction of pathogenesis-related proteins. In an ecological context the findings reveal that belowground neighbor detection may occur independently of resource depletion, allowing for a time advantage for the root to prepare for potential interactions.

## Introduction

Information on neighboring organisms is crucial to a plant, because neighbors are potential interaction partners both in competition and facilitation (Cahill et al., 2010; Faget et al., 2013). Aboveground mechanisms of plant neighbor detection are well understood. The ratio of red to far-red wavelength bands is altered by neighboring plants through light transmission or reflection, and can therefore be perceived as a signal (Ballaré et al., 1990). By contrast, belowground mechanisms of neighbor perception are by far less understood and they are likely to be more complicated. Gersani et al. (2001) demonstrated that plants with root contact engaged in a “Tragedy of the Commons” even when resources per plant are kept constant. Hence, it became clear that roots can perceive each other and as a consequence the plants increase allocation to roots. This finding triggered intense research on root-root interactions that comprises several related ideas and research topics namely (i) distinction of kin and stranger roots (stranger recognition) (ii) distinction of own and foreign roots (self/non-self recognition) (iii) perception of the presence/absence of neighbor roots (neighbor perception). In all of these research directions there are reports on plastic responses of interacting roots fostering those attributes that can increase belowground competitive ability. Yet, there are also many reports that do not confirm such findings, so that there are several connected topics that need to be followed up for functional mechanisms:

1. Stranger recognition, i.e., differentiation of close or distant genetic relations, was demonstrated for a number of different species. *Cakile edentula* var. *lacustris* increased allocation to roots when sharing a pot with strangers as opposed to siblings (Dudley and File, 2007; Bhatt et al., 2011). *Impatiens pallida* was found to vary in above-ground traits depending on neighbor identity, but only when root contact was given (Murphy and Dudley, 2009). *Arabidopsis thaliana* responded differentially to root exudates from siblings and strangers by forming more lateral roots when treated with exudates from stranger genotypes (Biedrzycki et al., 2010). Likewise, root exuded proteins and metabolites were found to differ depending on neighbor identity (Badri et al., 2012). Root exudates are therefore seen as possible mediators of stranger recognition (Bais et al., 2006; Badri and Vivanco, 2009; Biedrzycki and Bais, 2010). Indeed some components of plant exudates have already been found to be perceived by some specialized plant species (e.g., Strigolactones perceived by parasitic plants, Koltai et al., 2012). Likewise, Arabionogalactan proteins from root exudates are involved in many interactions of plant roots (e.g., with microbes), though any role of these in plant-plant interactions has as yet not been proven (Nguema-Ona et al., 2013). However, other studies reported that stranger recognition is a rather uncommon phenomenon (Milla et al., 2012; Lepik et al., 2012). Furthermore, *A. thaliana* plants in soil culture were not affected by relatedness to their neighbor (own or stranger genotype) and not even whole genome transcriptome analysis found differences related to neighbor genotypes (Masclaux et al., 2010).
2. In self/non-self recognition there are variable findings concerning reactions and proposed mechanisms: Falik et al. (2003) found that split root peas produced less root biomass when interacting with own roots than with roots from other plants. Only part of this reaction could be attributed to physiological co-ordination, the other part was possibly due to allorecognition. In contrast to this, Semchenko et al. (2007) found that neighbor identity (same clone, different clone) did not influence root reactions neither in *Fragraria vesca* nor in *Glechoma hederacea*. Biedrzycki et al. (2010) found that *A. thaliana* developed shorter roots in hydroponic solution which previously contained a different genotype than in its own hydroponic solution. Since the application of a secretion inhibitor had no effect on this response, it was followed that this case of self/non-self recognition was not mediated by root exudates but must have been due to other mechanisms (Biedrzycki et al., 2010).

Neighbor perception in general is a well-known phenomenon (Callaway, 2002). Altered root placement due to the presence of neighboring roots are well-known indicators of belowground neighbor perception and usually feature avoidance reactions (root segregation, Schenk et al., 1999; Cahill et al., 2010). Bartelheimer et al. (2006) varied presence/absence and species identity of neighbors in a controlled field experiment and found that in the presence of a neighbor, horizontal root distribution was altered and roots were placed toward rather than away from the neighbor (root aggregation). It was followed that such root reactions increase competitive ability and would be triggered by cues other than resource depletion. A recent study by Masclaux et al. (2012) analyzed the transcriptomic outcome of a competition setup with *A. thaliana* allowing for both, intraspecific interaction and intense resource depletion. A number of differentially expressed genes were found enriched in gene networks involved in nutrient deficiency and biotic stress. In detail the experiment revealed that in competing roots especially genes involved in cation transport, sulphur compound metabolic processes, transport processes, and secondary metabolism were affected, as well as many genes responsive to plant hormones. From a list of gene sets responsive to various stresses, the same experiment found enrichments in sets responsive to nitrogen-, phosphorus-, or potassium-starvation, cold-, salt- and wounding-stress, as well as to interaction with different pathogens (Masclaux et al., 2012). On the other hand, Nord et al. (2011) found no evidence for altered root placement due to the presence/absence of neighbors in common bean and showed that all observed root reactions were mediated by resource availability.

Following the above considerations it is clear that root-root interactions are as yet unpredictable, and the observed modes of reactions are highly diverse. Especially the mechanisms of neighbor perception and distinction are unclear (De Kroon, 2007) and current explanations range from the perception of resource depletion over physiological integration, if present, to mediation by root exudates.

In this paper, we address the topic of interspecific neighbor perception by a presence/absence approach. In order to identify the root-morphological and gene-transcriptional outcome of root interactions, we combined a root interaction experiment and a transcriptome analysis. Single plants of *A. thaliana* were either grown solitarily (control) or in the presence of *Hieracium pilosella*, which was chosen for its competitive inferiority to *A. thaliana*, thus minimizing effects and intensity of resource depletion. Different to the setup chosen by Masclaux et al. (2012), we vary the presence of a heterospecific instead of a conspecific neighbor.

The underlying hypotheses are

1. Heterospecific neighbor roots (represented by the weak competitor *H. pilosella*) induce characteristic modifications in the *A. thaliana*-transcriptome, which go beyond effects attributable to resource depletion.
2. The alterations in transcript levels between roots of solitary plants and those grown with a neighbor provide information on how *A. thaliana* reacts to the presence of heterospecific neighbor roots.

## Methods

### Experimental strategy

We used *A. thaliana* as target species to examine impacts of neighbors on both root morphological traits and genome-wide gene transcription. While intraspecific approaches have been reported before (Masclaux et al., 2012), *Hieracium pilosella* was used as an interspecific neighbor species. The species was chosen for the following reasons: gene transcription is strongly dependent on plant size, ontological stage and on environmental factors (von Tienderen et al., 1996). We minimized such factors co-varying with the presence/absence of a neighbor by using the weak competitor *Hieracium pilosella* challenging *A. thaliana*. The reasoning of this is that a weak competitor has little impact on the biomass of the target plant and produces a low degree of resource depletion (cf. Müller and Bartelheimer, 2013). In addition, *H. pilosella* is a rosette plant, so shading did not take place in this experiment. Both species naturally co-occur in European dry sandy grasslands [Sedo-Scleranthetalia, Corynephoretum (Hegi, 1986)]. The two treatments (with/without neighbor) were cultivated with an *n* = 18 to give an *n* = 9 for morphological traits and an *n* = 9 for transcriptional traits.

### Plant culture

**Figure 1:**
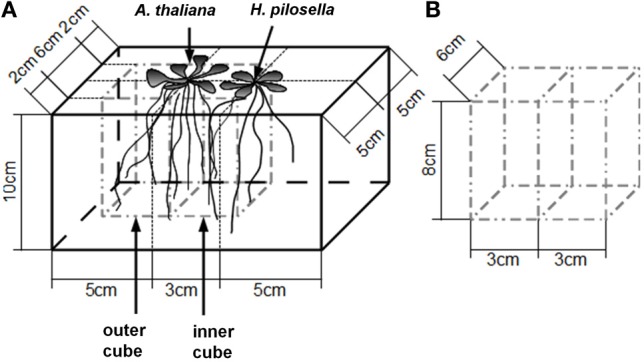
**Sketch of the method to assess *Arabidopsis thaliana* root reactions to the presence/absence of neighbors. (A)**, Plant container indicating the arrangement of plants and dimensions of the inner and the outer cubes cut at harvest. The position of the “neighbor” can either be filled by a plant of *Hieracium pilosella* or remain unfilled (control). Note that the central cut line is situated directly under the *A. thaliana* stem. **(B)**, Dimensions of the two-chambered open-bottom steel frame used to cut the cubes.

**Figure 2:**
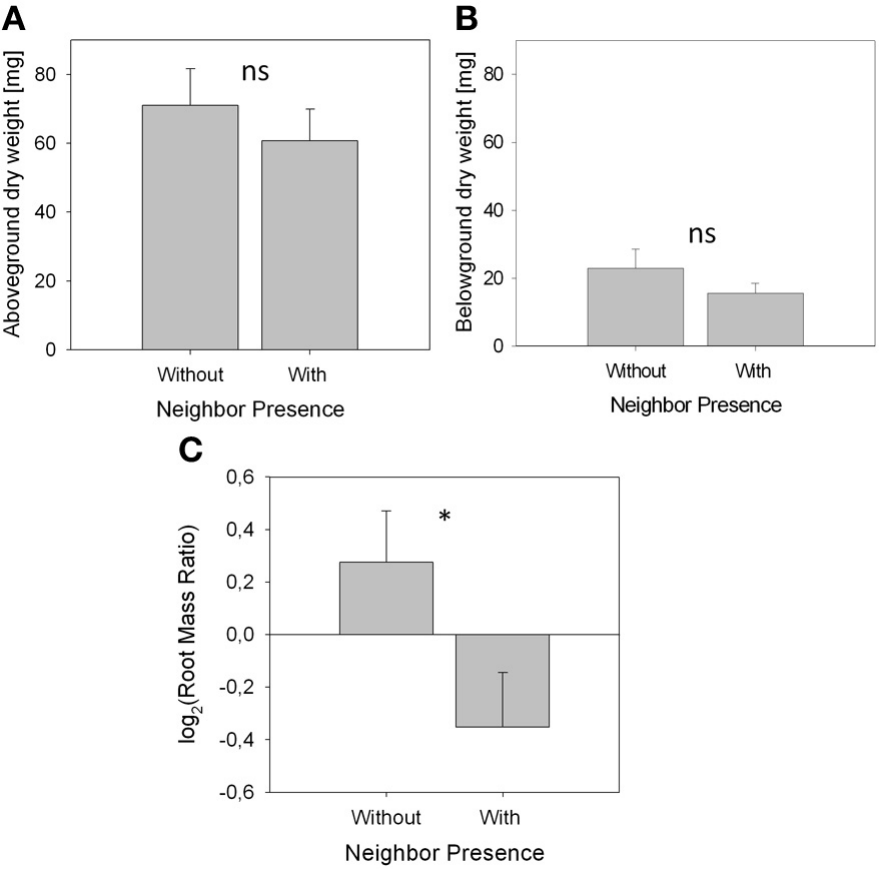
**Biomass parameters and root distribution (means ± SE) of *A. thaliana* without neighbor (control, *n* = 11) and with neighbor present (*n* = 9)**. Asterisks denote statistically significant differences in *t*-tests with ^*^p < 0.05; ns, not significant. **(A)**, Above-ground biomass. **(B)**, Belowground biomass. **(C)**. Root distribution pattern represented by log_2_(RR) based on root dry weight.

The experiment was set up in a climate chamber (short day conditions: 8/16 h; 20/15°C; 50% relative humidity), where photon flux density was 132 ± 3 μmol m^−2^ s^−1^ (mean ± SE measured at 18 evenly distributed points directly above the pots). Both *A. thaliana* (Col-0) and *H. pilosella* (wild collection near the village of Bad Laer, Lower Saxony, Germany) were germinated on potting soil (Einheitserde Classic, Pikiererde CL T, Einheitserde- und Humuswerke Gebr. Patzer GmbH & Co.KG, Simtal-Jossa, Germany), pricked out as seedlings and grown for 10 days on a sand/potting soil substrate (2 parts sand/1 part potting soil). Equally developed plants with four leaves were used for the experiment, where plants were cultured in rectangular pots (13 × 10 × 10 cm l/w/h, compare Figure 1A) filled with quartz sand (1.7 kg dry weight; max. grain size 0.7 mm) as substrate. *A. thaliana* seedlings were planted at defined positions either as control or with a plant of *H. pilosella* 3 cm apart (interaction treatment) (Figure 1A). 100 ml of nutrient solution were added to the cache pots on a weekly basis (875 μM NO^−^_3_; 125 μM H_2_PO^−^_4_; 125 μM K^+^, 250 μM Ca^2+^; 65 μM Mg^2+^; 65 μM SO^−^_42_; 7.5 μM Fe^3+^; 22.5 μM Cl^−^; adjusted to pH = 6.0) and additional deionized water (50–100 ml) was supplied according to consumption. By this fertilizer regime we kept resources constant per pot, though not per plant (compare Bartelheimer et al., 2006; Fang et al., 2013 for similar setups with additive designs on interspecific or intergenotypic interactions), taking into account the competitive inferiority of *H. pilosella* to *A. thaliana* (compare Figure 2 and Müller and Bartelheimer, 2013) to minimize effects of resource depletion. Plants were harvested after 48–50 days after planting and before shoot buds were visible.

**Figure A1:**
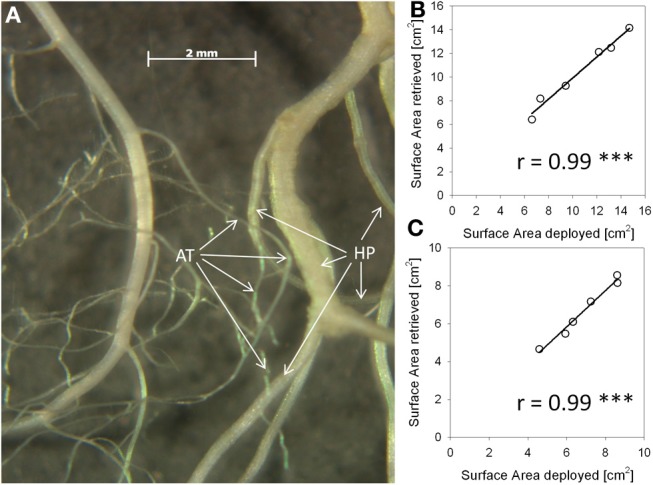
**Method validation for distinction of roots of the two experimental species**. Both in the experiment and in this validation setup roots were distinguished by visual inspection on the grounds of root morphological features. **(A)**: Photograph of water-floating roots of *Arabidopsis thaliana* (AT) and *Hieracium pilosella* (HP). **(B)**: Correlation results in *A. thaliana* root retrieval test of deployed vs. retrieved root fragments before and after thoroughly mixing them with root fragments of *H. pilosella*. Roots were placed in a transparent water basin, scanned and analysed for root surface area by WinRhizo imaging software. **(C)**: Corresponding results for *H. pilosella*. The number of replicates was *n* = 6.

We measured horizontal root placement in two adjacent cubes (3 × 6 × 8 cm l/w/h, compare Figure 1B) by cutting the soil beneath the *Arabidopsis* plants in two equal halves. These halves (termed inner and outer cube in the following, Figure 1), represented the soil sphere close to the neighbor plant, if present, and the soil sphere on the distant side, respectively. For cutting we used a sharpened metal frame (dimensions according to Figure 1B), which was pushed into the soil after cutting off the leaf rosettes. Roots from the cubes as well as from the remainder in the pot were washed out of the sand by use of a 1 mm sieve. Roots were assigned to species on the basis of differences in color, general morphology, and diameter. *H. pilosella* roots are silverish to yellowish, somewhat wrinkled and comparatively thick and can be easily distinguished from the white, unwrinkled and very fine roots of *Arabidopsis* by visual inspection. Validation of accuracy of the method by repetition examples as well as a photograph of both species' roots are available as supplemental material (Figure A1). Roots were spread on a glass recording tray and scanned with 300 dpi in gray shades with an EPSON Perfection V700 Photo scanner with transparency lighting system (Seiko Epson Corporation; Suwa, Nagano; Japan). Scans were analyzed with WinRhizo (V. 2008a Pro; Regent Instruments Canada Inc.; Ottawa; Canada) with a threshold value of 230 for background distinction and filters for objects smaller than 0.001 cm^2^ and with a length to width relation smaller than 6.0. All plant parts were oven-dried at 70°C to constant weight.

Horizontal root distribution was expressed as the log2-transformed ratio of roots in the outer and inner cube (compare Figure 1) with

$$\log2RR=\log_{2}(R_{\text{inner}}/R_{\text{outer}})$$

with log2*RR*: log2-transformed root ratio; *R*_inner_: root parameter in the inner cube (toward the neighbor); *R*_outer_: root parameter in the outer cube (away from the neighbor).

The log2*RR* was applied as it is symmetric around zero, meaning that during calculation of mean values, a particular ratio makes the same numerical contribution as its reciprocal value, which would not be the case for untransformed ratios.

### Root sampling for RNA extraction

To minimize the circadian impacts on gene activity sampling started 3 h after the climate chamber switched to “day-conditions.” From the nine individual plants per treatment, samples of three plants each were pooled to minimize effects of individual variation and to give a total *n* = 3 of independent biological replicates for molecular analyses. Soil cubes were cut as described above, but only the inner cube (position close to the neighbor if present, compare Figure 1) was used further. Cubes of the three respective plants were pooled in a 1 mm sieve and washed out of the sand. Roots were suspended in water and thoroughly assorted by species. *Arabidopsis* roots were cut from the tap-root and stored at −80°C for further use. Processing time per pooled sample was exactly 9 min each. Samples requiring less time, especially controls, where root sorting did not apply, were left to stand until the 9 min were up, to prevent effects of different harvest duration on transcription.

### PCR-based screening for *Phytophtora* contaminations

During data analysis (see below) a high degree of resemblance in transcriptome response to roots challenged by *Phytophthora spec*. made a screen for contamination necessary. Absence of any *Phytophthora*-contamination was tested by PCR using genus-specific primers FMPh-8b: AAAAGAGAAGGTGTTTTTTATGGA and FMPh-10 b: GCAAAAGCACTAAAAATTAAATATAA.

### Genechip microarray analysis

Tissues were homogenized with 1.4 mm ceramic beads for 30 s at 6,000 rpm using the Precellys Homogenizer (PEQLAB, Erlangen, Germany), followed by total RNA purification with RNeasy Mini columns (Qiagen, Hilden, Germany) and an Agilent 2100 bioanalyzer quality assessment (Agilent Technologies, Palo Alto, USA). Gene expression profiles were determined by Arabidopsis ATH1 Genome Arrays according to the GeneChip 3′ IVT Express Kit Manual (Affymetrix, Santa Clara, USA). Two hundred and fifty nanogram of total RNA were used to generate double-stranded cDNA and subsequently Biotin-labeled aRNA. Following fragmentation, aRNA products were hybridized to the array for 16 h at 45°C in a rotating chamber. Hybridized arrays were washed and stained in an Affymetrix Fluidics Station FS450, and the fluorescent signals were measured with an Affymetrix GeneChip Scanner 3000-7G. Tissue homogenization, RNA purification and sample processing were performed at an Affymetrix Service Provider and Core Facility, “KFB—Center of Excellence for Fluorescent Bioanalytics” (Regensburg, Germany).

### Microarray data analysis

The MAS5 algorithm of the Affymetrix Command Console Software (AGCC) was used for Single Array Analysis. A global scaling strategy was employed by setting the average signal intensities of all arrays to a target value of 500. All detectable expressed genes were defined using P-, M- or A-calls. Baseline comparison and significance analysis (unpaired *t*-test) were performed in Microsoft Excel.

Significantly regulated genes had to meet the following criteria: (a) a *p*-value smaller than 0.05 and (b) expressed (P-call) in at least two of the six samples.

Microarray data was deposited at the ArrayExpress repository under accession number E-MTAB-1582.

### Data processing

Relative expression values were calculated and expressed as log_2_-transformed mean signal ratios. Functional category scoring (Table 2, Figure 6) was implemented using MapMan software (Usadel et al., 2005), where all non-significant log_2_(mean signal ratios) were set to zero and Wilcoxon Rank Sum tests with Benjamini Hochberg correction were applied.

A signature analysis using Genevestigator software (Hruz et al., 2008) was carried out to depict microarray studies resembling the microarray at hand (termed neighbor-perception microarray in the following). From the list of significantly altered gene-products with at least two P-calls, the 20 genes with the highest as well as the 20 genes with the most negative log_2_(mean signal ratios) were used to represent the neighbor-perception-array. The Genevestigator database was narrowed down to include solely studies in lateral roots of Arabidopsis wildtype, resulting in a total of 537 transcriptome studies. Manhattan Distance based on the 40 named transcripts was used as a measure to determine the relative similarity of particular arrays from this database to the neighbor-perception-array. For this, Genevestigator software relates an absolute similarity value (in our case based on the 40 mentioned genes) to an average similarity gained over all included experiments. More precisely, if the similarity S_i_ is defined as 1/d_i_ with d_i_ the distance of category i to the signature then the relative similarity RS of a category c is calculated following the formula

$$RS_{c}=S_{c}/1/N\Sigma_{i\;\epsilon I}S_{i}$$

with *RS*, relative similarity; *S*, absolute similarity; *c*, considered category.

Higher values in relative similarity thus indicate higher similarity relative to average similarity (according to documentation on www.genevestigator.com; accessed Feb. 26, 2013).

All other statistics were carried out using SPSS 19 software (SPSS, Chicago, IL, USA).

## Results

### The presence of *H. pilosella* roots induces root segregation in *arabidopsis*

Biomass of both above- and belowground plant parts as well as total biomass were slightly but not significantly reduced when a neighbor was present (Figures 2A,B; Table 1). Root distribution varied significantly between treatments with control plants placing more of their root biomass in the center of the pot [log_2_(Root Mass Ratio) > 0] while plants exposed to neighbors placed more root biomass toward the margin of the pot [log_2_(Root Mass Ratio) < 0; Figure 2C]. Measures of root length and root surface area reacted in accordance with root biomass distribution (Table 1), indicating that *A. thaliana* placed its roots preferably away from the neighbor (root segregation). Root diameter was overall higher in the neighbor treatment, which was especially the case in roots that were placed away from the neighbor (outer cube) but less so in roots that were close to the neighbor (Table 1).

**Table 1:** **Biomass and root morphological traits (means ± SE) of *A. thaliana* in control (*n* = 11) and neighbor contact treatment (*n* = 9)**.

	**Root fraction**	**Control**	**With neighbor**	***p*-Value**	**Effect size *r***
Total biomass [g]	–	94.04 ± 16.01	76.31 ± 11.92	>0.05	0.20
Root / shoot ratio	–	0.29 ± 0.03	0.24 ± 0.03	>0.05	0.21
Root length [cm]	inner cuboid	548.72 ± 49.71	389.99 ± 74.13	>0.05	0.40
	outer cuboid	459.78 ± 62.06	515.37 ± 87.01	>0.05	0.12
	Σ	1008.50 ± 95.43	905.37 ± 154.91	>0.05	0.14
	**log_2_(ratio)**	**0.33** ± **0.20**	−**0.46** ± **0.19**	0.013^*^	**0.55**
Root surface area [cm^2^]	inner cuboid	37.93 ± 3.58	27.96 ± 5.41	>0.05	0.35
	outer cuboid	30.43 ± 3.98	37.51 ± 6.41	>0.05	0.22
	Σ	68.36 ± 6.10	65.47 ± 11.35	>0.05	0.06
	**log_2_(ratio)**	**0.38** ± **0.23**	**−0.50** ± **0.20**	0.010^*^	**0.56**
Root diameter [mm]	inner cuboid	0.2193 ± 0.0047	0.2285 ± 0.0048	>0.05	0.30
	outer cuboid	0.2114 ± 0.0022	0.2354 ± 0.0066	0.006^**^	**0.74**
	mean	0.2153 ± 0.0029	0.2320 ± 0.0056	0.021^*^	**0.63**

‘Inner and outer cube’ are soil volumes cut from positions below the plants according to Figure 1. Asterisks denote statistically significant differences in T-tests with

* p < 0.05 and

** *p < 0.01*.

*Effect sizes were calculated as Pearson's correlation coefficient r with r ≥ 0.1 showing a small, r ≥ 0.3 a medium and r ≥ 0.5 a large effect of the treatment on corresponding variable (Cohen, 1988)*.

The part experiment that was sampled for transcriptome analysis (nine plants per treatment) could not be analyzed for root dry weight data, but above-ground biomass was assessed. As in the part experiment used to infer biomass and root distribution data, above-ground biomass was slightly but not significantly reduced when a neighbor was present (control: 80.86 ± 5.49 mg; neighbor treatment: 67.79 ± 3.95 mg; mean ± SE for *n* = 9; ns in *t*-test with *p* = 0.071; data not shown).

### The transcriptome analysis reveals “Biotic Interactions” as a major mechanism of neighbor perception

Amounts of gene transcripts as measure of gene activity were examined using ATH1-microarrays of *Arabidopsis* roots exposed to roots of *H. pilosella* and of controls without neighbor roots. In a total of 22,810 expressed genes, we found 797 and 652 significantly induced and repressed transcripts, respectively, cf. Table S1, meaning that neighbor contact affected 6.35% of examined genes.

**Figure 3:**
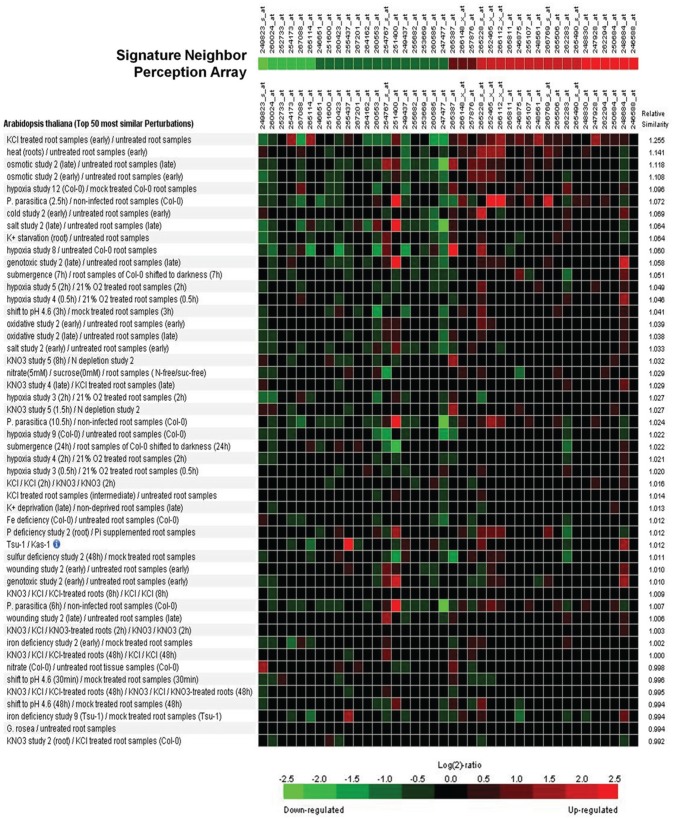
**Signature analysis between the neighbor-perception-microarray and transcriptome studies on various perturbations from the Genevestigator database**. From the list of significantly altered gene-products with at least two P-calls, the 20 genes with the highest and the 20 genes with the most negative induction factor log2(mean signal ratio) were used to represent the neighbor-perception-array. The Genevestigator database was narrowed down to include solely studies in lateral roots of Arabidopsis wildtype, resulting in a total of 537 transcriptome studies. Green squares represent repression of the according gene in a particular array study, red squares represent induction.

**Figure 4:**
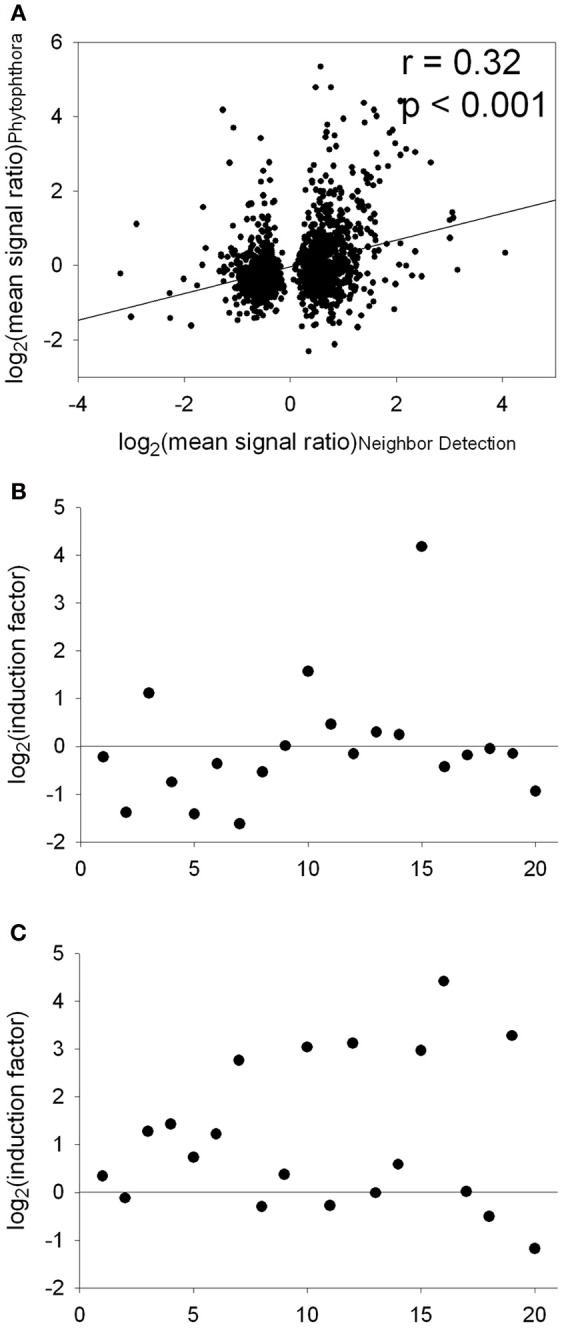
**Comparison of transcript abundance in the “neighbor perception experiment” and the “*Phytophthora* interaction study” [Attard et al., 2010; data downloaded from the Gene Expression Omnibus epository of the National Center for Biotechnology Information (http://www.ncbi.nlm.nih.gov/)]. (A)**, Correlation of log_2_(mean signal ratio) comprising all genes significantly regulated in the “neighbor perception experiment.” **(B)**, Plot of the 20 genes most strongly repressed in the “neighbor perception experiment” vs. the according induction factors from the “*Phytophthora* interaction study.” **(C)**, Plot of the 20 genes most strongly induced in the “neighbor perception experiment” vs. the according induction factors from the “*Phytophthora* interaction study.”

**Figure 5:**
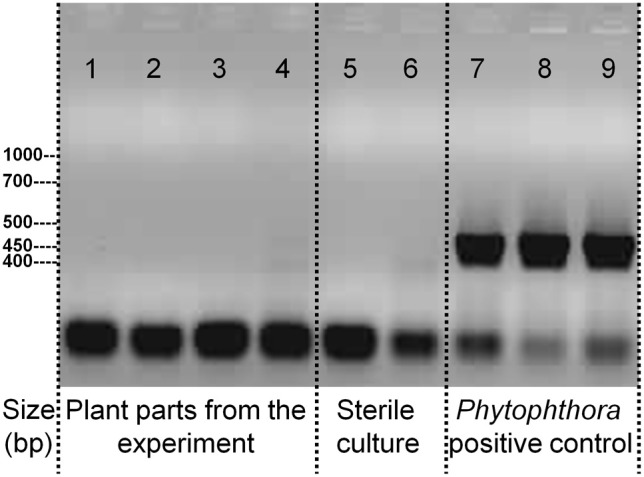
**PCR-test for the presence of DNA specific to the parasitic Oomycet genus *Phytophthora* in the experiment**. Applied PCR-primers are specific to the entire genus. Displayed samples are from the experiment itself with 1. *A. thaliana* roots, grown in presence of *H. pilosella*, 2. *A. thaliana* leaf, grown in presence of *H. pilosella*, 3. *H. pilosella* roots, 4. *A. thaliana* roots, grown in absence of *H. pilosella*; or are samples raised under sterile conditions with 5. *A. thaliana* roots, 6. *H. pilosella* roots; or are inoculated samples from separate cultures with 7. *Phytophthora* (pure culture), 8. *A. thaliana* roots, 9. *H. pilosella* roots.

A signature analysis (Figure 3) between the present neighbor-perception microarray, represented by the 20 most strongly induced plus the 20 most strongly repressed gene-transcripts, and microarrays from the collection of Genevestigator microarray data base (Hruz et al., 2008) revealed considerable similarity to a number of perturbations. These included exposure of *Arabidopsis* roots to zoospores of the parasitic Oomycet *Phytophthora parasitica* for 2.5 h (cf. Attard et al., 2010) as well as exposure to KCl, heat, osmotica (in this case mannitol), hypoxia, and potassium starvation (Figure 3). A more detailed analysis between the neighbor-perception microarray and the named microarray by Attard et al. (2010) revealed that the overall transcriptomic response correlates highly significantly (*r* = 0.32; *p* < 0.001) (Figure 4A). It is also pinpointed that among the named 20 most strongly induced gene-transcripts from the neighbor-perception microarray 13 are also induced in the microarray by Attard et al. (2010) while only five minor mismatches are found (Figure 4C). In repressed gene-transcripts twelve matches were found as opposed to six mismatches (Figure 4B). To test for any contamination of the plant material used in this study by the plant pathogen *Phytophthora* sp. a PCR-based *Phytophthora* screen was performed (Figure 5). This showed that no infection by any *Phytophthora* species was detectable. It is thereby indicated that the observed similarity in gene expression to *Phytophthora* exposition studies is not due to an actual infection by *Phytophtora* pathogens, but to similar plant reactions to root neighbors (and / or associated microorganisms.

**Figure 6:**
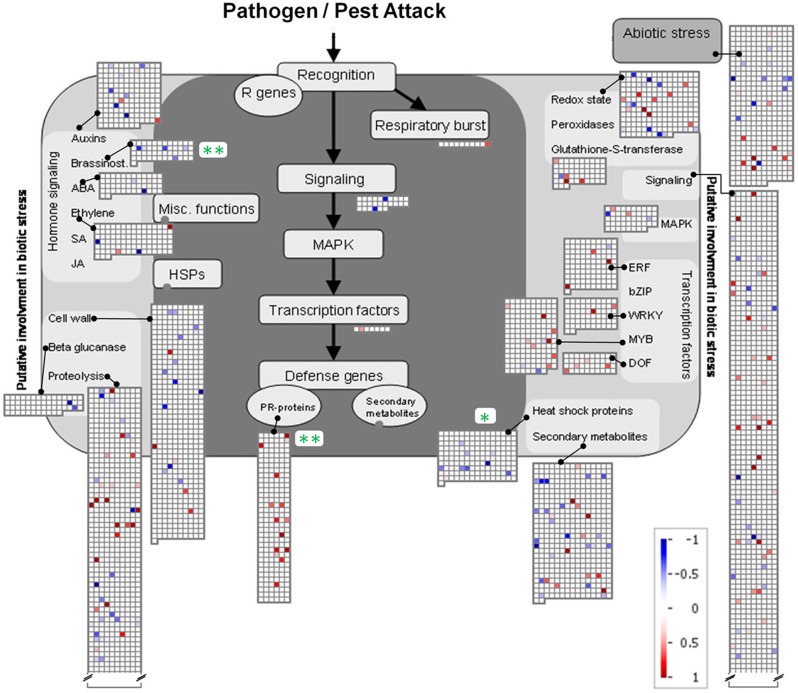
**MapMan depiction of gene regulations in functional categories associated with Ćbiotic stress' in *A. thaliana* roots exposed to *H. pilosella***. Asterisks denote significantly regulated functional (sub-)categories following Wilcoxon Rank Sum statistics (Benjamini Hochberg corrected) with ^*^p < 0.05; ^**^p < 0.01. Color-coded squares represent single significantly regulated genes; blue: repressed transcription; red: induced transcription. Note that functional categories with >2 sign. regulated genes are mostly not displayed.

To further analyze the similarity between root-root contact and root-pathogen contact a MapMan analysis (Usadel et al., 2005) concerning functional (sub-)categories with involvement in pathogen and pest attack was carried out (Figure 6). Three categories with significant regulation were identified. Two were significantly repressed (“Brassinosteroids” in the category of “Hormone Signaling” and “Heat Shock Proteins”). Highly significant induction was found for “PR-proteins”, where 14 significantly induced and no repressed gene products were found. Further significant categories were not detected; however, when taken together the four sub-categories subsumed under “Transcription Factors” comprise a considerable number of induced genes products. Two of these belong to the WRKY domain transcription factor family (*At2g30250* and *At1g29280*), eight to the MYB domain transcription factor family (*At5g49620*, *At4g33450*, *At4g09460*, *At3g04030*, *At3g55730*, *At3g27220*, *At1g17950*, *At3g09370*), and four to the MYB-related transcription factor family (*At5g47390*, *At5g01200*, *At1g74840*, *At3g49850*) (Figure 6). A further three induced gene products of transcription factors belong to the ethylene-responsive element binding protein family (*At2g23340*, *At3g16770*, *At4g25480*), and one to the C2C2(Zn) DOF zinc finger family (*At3g50410*) (Figure 6). A possible involvement of these transcription factors in the activation of PR-proteins is thereby indicated, and in fact, at least one of these induced transcription factors [*At3g16770*, *ERF72*, log_2_(signal ratio) = 1.56] has previously been shown to induce certain pathogen responsive genes (Ogawa et al., 2005). Likewise, the involvement of transcription factors of the WRKY family (see above) in pathogen response has repeatedly been described [reviewed by Rushton et al. (2010)].

General transcriptional responses were evaluated on the basis of functional categories and sub-categories (Table 2). The significant regulations of functional categories including all sub-categories were analyzed on the basis of the induction and repression (positive and negative log_2_-ratios) of all elements assigned to the respective functional (sub-)category according to the MapMan system [(sub-)BIN, Table 2]. Most BINs and subBINs were not significantly regulated, a considerable number was repressed and a smaller number was induced. The repressed BINs included OPP, TCA, and lipid metabolism. The functional category of photosynthesis (PS) is divided into the induced subBINs connected to light reaction and two repressed subBINs in photorespiration and Calvin cycle. Plastids in roots conduct no light reaction, and 13 out of 19 induced genes assigned to PS.lightreaction were plastid-encoded. Without light such plastid encoded genes can still be transcribed to considerable amounts, but the translation of the resulting mRNA is highly dependent on light and the biogenesis of plastids to chloroplasts. In roots, the according mRNA is therefore not translated into proteins (Mayfield et al., 1995; Spermulli, 2000). Some functional categories connected to cell energy status were repressed, e.g., OPP, TCA, and “Acetyl CoA carboxylation” in “lipid metabolism.” The few induced BINs include “Pathogenesis Related Proteins” in “Biotic Stress” as well as “Lipids” in the BIN “Signaling.”

**Table 2:** **Significantly regulated functional categories**.

**BIN code**	**BIN designation**	**Direction of regulation**	**Elements**	**Number of sig. induced genes**	**Number of sig. repressed genes**
1.1	PS.lightreaction	Induced^***^	136	19	0
1.1.1	PS.lightreaction. Photosystem II	Induced^**^	55	8	0
1.1.1.2	PS.lightreaction. Photosystem II.PSII polypeptide subunits	Induced^***^	44	8	0
1.1.40	PS.lightreaction. Cyclic electron flow-chlororespiration	Induced^**^	8	3	0
1.1.6	PS.lightreaction.NADH DH	Induced^**^	10	3	0
1.2.4	PS.photorespiration.glycine cleavage	Repressed^*^	6	0	2
1.3	PS.calvin cyle	Repressed	31	0	6
7	OPP	Repressed^**^	31	0	5
8	TCA / org. transformation	Repressed^***^	76	1	11
8.1	TCA / org. transformation.TCA	Repressed ^***^	41	0	8
8.1.1	TCA / org. transformation.TCA.pyruvate DH	Repressed^*^	13	0	3
8.1.1.1	TCA / org. transformation.TCA.pyruvate DH.E1	Repressed^**^	5	0	2
11.1.1	Lipid metabolism.FA synthesis and FA elongation.Acetyl CoA Carboxylation	Repressed^*^	7	1	3
13	Amino acid metabolism	Repressed^***^	225	1	20
13.1	Amino acid metabolism.synthesis	Repressed^***^	153	0	14
13.1.2	Amino acid metabolism.synthesis.glutamate family	Repressed^**^	8	0	3
13.1.2.3	Amino acid metabolism.synthesis.glutamate family.arginine	Repressed^***^	7	0	4
13.1.3	amino acid metabolism.synthesis.aspartate family	Repressed^*^	39	0	6
13.1.3.4	Amino acid metabolism.synthesis.aspartate family.methionine	Repressed^*^	20	0	4
16.1.2	Secondary metabolism.isoprenoids.mevalonate pathway	Repressed^***^	16	0	5
16.5.1	Secondary metabolism.sulfur-containing.glucosinolates	Repressed^*^	54	1	7
16.5.1.1	Secondary metabolism.sulfur-containing.glucosinolates.synthesis	repressed^**^	31	1	7
16.5.1.1.4	Secondary metabolism.sulfur-containing.glucosinolates.synthesis.shared	Repressed^***^	3	0	2
17.2.1	Hormone metabolism.auxin.synthesis-degradation	Repressed^**^	10	0	3
17.3	Hormone metabolism.brassinosteroid	Repressed^**^	49	0	6
17.3.1	Hormone metabolism.brassinosteroid.synthesis-degradation	Repressed^**^	31	0	5
17.3.1.2	Hormone metabolism.brassinosteroid.synthesis-degradation.sterols	Repressed^***^	19	0	5
17.3.1.2.2	Hormone metabolism.brassinosteroid.synthesis-degradation.sterols.SMT2	Repressed^***^	3	0	2
20.1.7	Stress.biotic.PR-proteins	Induced^**^	203	13	0
20.2.1	Stress.abiotic.heat	Repressed^*^	151	1	9
21.99	Redox.misc	Repressed^*^	6	0	2
23	Nucleotide metabolism	Repressed^*^	157	3	12
23.1.2	Nucleotide metabolism.synthesis.purine	Repressed^*^	15	0	3
23.4.1	Nucleotide metabolism.phosphotransfer and pyrophosphatases.adenylate kinase	Repressed^*^	6	0	2
25	C1-metabolism	Repressed^***^	33	0	7
25.1	C1-metabolism.glycine hydroxymethyltransferase	Repressed^*^	6	0	2
29	Protein	Repressed^**^	3123	109	133
29.1.20	Protein.aa activation.phenylalanine-tRNA ligase	Repressed^***^	3	0	2
29.2	Protein.synthesis	Repressed^***^	515	19	53
29.2.1	Protein.synthesis.ribosomal protein	Repressed^***^	371	14	36
29.2.1.1.1.1	Protein.synthesis.ribosomal protein.prokaryotic.chloroplast.30S subunit	Induced^*^	25	5	1
29.2.1.1.3.1	Protein.synthesis.ribosomal protein.prokaryotic.unknown organellar.30S subunit	Repressed^*^	14	1	4
29.2.1.2	Protein.synthesis.ribosomal protein.eukaryotic	Repressed^***^	236	1	25
29.2.1.2.1	Protein.synthesis.ribosomal protein.eukaryotic.40S subunit	Repressed^*^	88	0	7
29.2.1.2.2	Protein.synthesis.ribosomal protein.eukaryotic.60S subunit	Repressed^***^	148	1	18
29.2.1.2.2.19	Protein.synthesis.ribosomal protein.eukaryotic.60S subunit.L19	Repressed^**^	4	0	2
29.2.1.2.2.57	Protein.synthesis.ribosomal protein.eukaryotic.60S subunit.L7A	Repressed^**^	5	0	2
29.2.2.50	Protein.synthesis.misc ribosomal protein.BRIX	Repressed^*^	6	0	2
29.2.3	Protein.synthesis.initiation	Repressed^**^	84	3	12
29.5.11.20	Protein.degradation.ubiquitin.proteasom	Repressed^***^	53	0	13
29.5.11.3	Protein.degradation.ubiquitin.E2	Induced^*^	37	6	1
29.5.2	Protein.degradation.autophagy	Induced^*^	20	4	0
29.5.3	Protein.degradation.cysteine protease	Induced^*^	87	9	1
30.9	Signaling.lipids	Induced^***^	5	4	0
30.99	Signaling.unspecified	Repressed^*^	7	0	2
35	Not assigned	Induced^***^	7639	286	139
35.1.1	Not assigned.no ontology.ABC1 family protein	Induced^**^	11	3	0
35.2	Not assigned.unknown	Induced^***^	5386	208	89
35.3	Not assigned.disagreeing hits	Induced^*^	63	7	1

Asterisks denote statistically significant differences in Wilcoxon Rank Sum tests (Benjamini Hochberg corrected) with

* p < 0.05,

** p < 0.01,

*** *p < 0.001*.

*BIN code and BIN designation are numerical codes and short descriptions of hierarchically organized functional categories according to the MapMan tool (Usadel et al., 2005)*.

### Comparison to intraspecific competition

**Figure 7:**
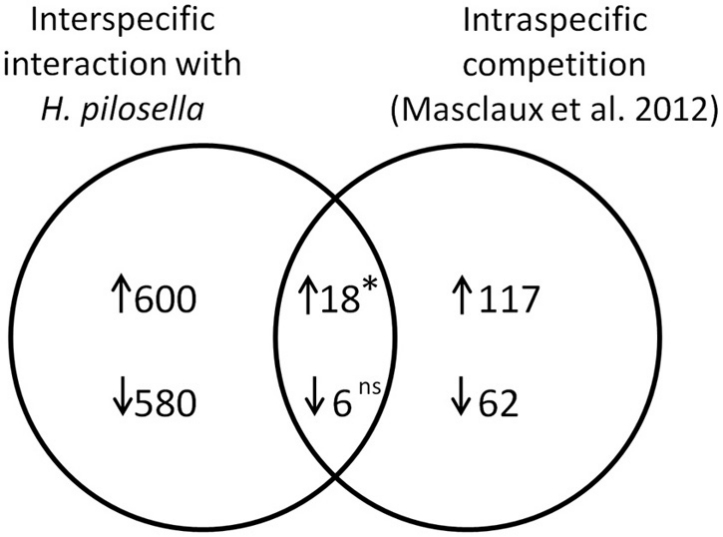
**Venn diagram with numbers of overlapping and non-overlapping differentially expressed genes in a comparison of *A. thaliana* roots challenged by interspecific interaction with *H. pilosella* (data from this paper) to *A. thaliana* roots challenged by intraspecific competition (Masclaux et al., 2012)**. Both experimental setups are additive designs (presence/absence of neighbors), while density of neighbors (if present) and impacts on biomass in examined plants is much higher in Masclaux et al. (2012). The overall number of analyzed transcript IDs (mutually utilized in both studies) was *N* = 13150. ↑, induced genes; ↓, repressed genes. The asterisk indicates a significantly higher value than numerical expectation from a 2^*^2table (cf. Nemhauser et al., 2006) with ^*^p < 0.05 in Chi^2^-Test; ns, not significant.

Interestingly, a recent study by Masclaux et al. (2012) examined the transcriptional outcome of intraspecific competition in roots. Our comparison included all genes that were mutually evaluated in both arrays (*N* = 13, 150), where numbers were reduced especially by cases with too few presence calls in our array. The number of genes exclusively induced in our array was *N* = 600, in the array by Masclaux et al. (2012) it was *N* = 117, and the overlap was *N* = 18 (Figure 7). This number of overlapping induced genes was significantly higher than would be expected from pure proportionality, i.e., in the absence of any concordant reaction (value expected from pure proportionality would have been 6.34; *p* = 0.028). In the case of repressed genes our array contained *N* = 580 exclusive cases, the array by Masclaux et al. (2012) contained *N* = 62 exclusive cases and the number of overlapping genes was *N* = 6 [not significantly different from number expected from proportionality (3.03)].

The list of genes that are concordantly induced or repressed, respectively, in both of these arrays possibly reflects those genes in *Arabidopsis* roots that are in general responsive to interaction with other plants. Reactions to nutrient depletion might be included as a secondary effect of neighboring roots. The induced genes (Table 3) comprise two genes from the functional category “biotic stress-PR-proteins” (BIN 20.1.7) as well as two genes from the functional category “signaling.receptor kinases” (BIN 30.2). Both among the induced and in repressed genes (Tables 3, 4) we find entries that are involved in the regulation of transcription (BIN 27.3; RNA.regulation of transcription). Therefore, both in intraspecific competition (Masclaux et al., 2012) and in interspecific interaction with *H. pilosella* genes with functions in pathogen response are induced, while only a smaller common core of these is a true communality between the two setups.

**Table 3:** **List of induced transcripts regulated in concordance with transcriptional responses to intraspecific competition as examined by Masclaux et al. (2012)**.

**Gene information**				**Ratio (log2)**	
**Transcript ID**	**NAME and/or Description**	**BIN code**	**BIN name**	**Neighbor perception (this study)**	**Intraspecific competition (Masclaux et al., 2012)**
At1g71697	CHOLINE KINASE 1	11.3.2	Lipid metabolism.Phospholipid synthesis.choline kinase	0.60	0.80
At5g40170	RECEPTOR LIKE PROTEIN 54	20.1.7	Stress.biotic.PR-proteins	0.98	1.16
At1g72920	Toll-Interleukin-Resistance domain family protein	20.1.7	Stress.biotic.PR-proteins	0.77	3.66
At3g19010	2-oxoglutarate and Fe(II)-dependent oxygenase superfamily protein	21.2	Redox.ascorbate and glutathione	0.56	1.17
At2g29720	CTF2B / monooxygenase activity. oxidoreductase activity	26.7	Misc.oxidases-copper. flavone etc.	0.73	1.64
At5g47390	MYB-like transcription factor family protein	27.3.26	RNA.regulation of transcription.MYB-related transcription factor family	0.39	0.75
At3g59700	LECTIN-RECEPTOR KINASE 1 / Receptor kinase-like protein family	30.2.19	Signaling.receptor kinases.legume-lectin	0.71	1.54
At1g53440	Leucine-rich repeat transmembrane protein kinase	30.2.8.2	Signaling.receptor kinases.leucine rich repeat VIII-2	0.77	0.96
At5g61210	SOLUBLE N-ETHYLMALEIMIDE-SENSITIVE FACTOR ADAPTOR PROTEIN 33	31.4	Cell.vesicle transport	0.637	1.076
At1g59910	Actin-binding formin homology 2 family protein	35.1.20	Not assigned.no ontology.formin homology 2 domain-containing protein	0.358	1.919
At5g28630	Unknown protein with unknown function	35.1.40	Not assigned.no ontology.glycine rich proteins	0.482	2.681
At3g55840	Hs1pro-1 protein / ortholog of sugar beet HS1 PRO-1 2	35.2	Not assigned.unknown	1.166	3.441
At1g05340	Unknown protein with unknown function	35.2	Not assigned.unknown	1.060	0.933
At3g45730	Unknown protein with unknown function	35.2	Not assigned.unknown	0.972	1.38
At3g51890	Clathrin light chain protein	35.2	Not assigned.unknown	0.671	0.972
At2g28570	Unknown protein with unknown function	35.2	Not assigned.unknown	0.568	0.883
At2g42950	Magnesium transporter CorA-like family protein	35.2	Not assigned.unknown	0.426	0.783
At2g32210	Unknown protein with unknown function	35.2	Not assigned.unknown	0.291	2.342

**Table 4:** **List of repressed transcripts regulated in concordance with transcriptional responses to intraspecific competition as examined by Masclaux et al. (2012)**.

**Gene information**				**Ratio (log2)**	
**Transcript ID**	**Description (name)**	**BIN code**	**BIN name**	**Neighbor perception (this study)**	**Intraspecific competition (Masclaux et al., 2012)**
At5g10130	Pollen Ole e 1 allergen and extensin family protein	20.2.99	Stress.abiotic.unspecified	−0.951	−1.031
At5g62340	Plant invertase/pectin methylesterase inhibitor superfamily protein	26.18	Misc.invertase/pectin methylesterase inhibitor family protein	−1.218	−0.805
At3g49940	LOB (Lateral organ boundaries) DOMAIN-CONTAINING PROTEIN 38	27.3.37	RNA.regulation of transcription.AS2, lateral organ boundaries gene family	−0.939	−0.776
At3g04070	NAC domain containing protein 47	27.3.27	RNA.regulation of transcription.NAC domain transcription factor family	−0.334	−1.269
At5g60680	Unknown protein with unknown function	35.2	Not assigned.unknown	−0.359	−1.325
At3g19680	Unknown protein with unknown function	35.2	Not assigned.unknown	−0.431	−0.965

## Discussion

### Alteration of root distribution as avoidance of potential competition

We found that in the presence of a neighbor, *A. thaliana* places more roots toward the margin than toward the center of the pot (Table 1, Figure 2), i.e., the neighbor root is avoided by root segregation (Schenk et al., 1999). This reveals unambiguously that the neighbor plant was detected and had a high impact on root distribution with an effect size of *r* > 0.5 (Table 1), which allows us to further analyze the mechanisms of neighbor perception (see below). It also reveals that in this experiment *Arabidopsis* avoids intense root overlap and potential competition. A spectrum from avoidance to confrontation of neighbor root systems has been found for different species in different experiments (Schenk et al., 1999 for segregation, Bartelheimer et al., 2006; Semchenko et al., 2007 for aggregation). To discuss the question what was the cause of the observed root segregation when a neighbor was present, at least two non-exclusive answers are possible [also reviewed by Hodge (2012)]. The first possibility is the perception of the neighbor by mechanisms beyond resource depletion. The second involves resource depletion due to consumption by neighbor roots. As for the perception of neighbor roots, they may be attributed to physical contact (Mahall and Callaway, 1991) or to root exudates (Bais et al., 2006) or to associated microbial organisms and substances of microbial origin (Steenhoudt and Vanderleyen, 2000) (a detailed discussion on possible functional mechanisms is found in the paragraphs below). As for perception of local resource depletion as the second possible explanation (Schenk et al., 1999; Nord et al., 2011), it is known that roots often proliferate less, where resources are scarce and allocate more growth to where resource availability is higher (Gersani et al., 1998; Hodge, 2004). Indeed, some signs for moderate resource depletion were found, since the signature analysis (Figure 3) detected similarities between the neighbor-perception microarray and experiments on potassium starvation as well as (to a lower extend) on other nutrient deficiencies. Consequently, local resource depletion may well have played a role as cue for the presence of a neighbor in the study at hand. However, signs of resource competition like reduced plant biomass or affected root/shoot ratios were weak, as indicated by small effect sizes of *r* = 0.20 and 0.21, respectively, and non-significant (Figure 2, Table 1). It is thus unlikely that resource depletion alone caused the described reactions. In fact, a recent study by Cahill et al. (2010) on Abutilon theophrasti found resource availability and neighbor perception to act in concert with segregation occurring solely when both, neighbor presence and uniform distribution of resources, were given. In the case of our study, resource availability was not varied between treatments and depletion likely was low. Considering the results by Cahill et al. (2010) it is therefore well conceivable that *A. thaliana* in our study reacted in a similar information-integrating manner as *Abutilon theophrasti*: segregative root placement, when a neighbor is present and resources are relatively homogenously distributed.

Under natural conditions, root segregation as was found in our experiment is likely an essential strategy for annual species to maximize resource access. Detecting the neighbor early on will optimize this process, because this allows reactions even before the, often negative, interaction takes place. With neighbor perception as a prerequisite to optimize a plant's growth strategy, it is clear that we need to find out more about its mechanisms.

### Belowground neighbor perception and transcriptome analysis

We found that the presence of a neighbor led to a high number of differentially transcribed genes Table S1. This allows the application of bioinformatic approaches to the topic of interspecific neighbor perception. These analyses included the detection of significantly regulated functional categories as well as a signature analysis for similarity with a broad variety of microarrays that cover the impact both of biotic and abiotic environmental factors. In addition, our data allows for comparisons of differentially expressed genes to those found in previous studies on similar topics (Broz et al., 2008; Biedrzycki et al., 2011; Masclaux et al., 2012).

### Functional categories: pathogen response genes induced during neighbor perception

The most striking finding from the transcriptome analysis was the induction of genes coding for pathogenesis-related proteins (Table 2, Figure 6). These genes are known to respond to a number of different biotic stresses (Stintzi et al., 1993; Sels et al., 2008), but so far little is known about their role in plant neighbor perception. These PR proteins are a clear indication that not resource depletion but biotic signals mediated the detection of *H. pilosella* roots. We suggest that such biotic cues could be either the neighbor root itself, including its exudates, or it could be microorganisms associated to the neighbor root.

The presence of *Phytophthora* sp. itself was ruled out (Figure 5), which was pivotal after the detection of a high transcriptional similarity to a setup that challenged roots with *Phytophthora* oospores. Still, non-pathogenic microorganisms will have been present due to the non-sterile growth conditions of our setup. *H. pilosella* is a species strongly colonized by Vesicular Arbuscular Mycorrhiza (VAM) when raised on sand (personal observation), while *A. thaliana* is non-mycorrhizal. VAM as an agent of neighbor perception is a possibility, though support from the literature for this notion is weak: VAM are known to repress rather than induce genes coding for PR-proteins (Ginzberg et al., 1998; Shaul et al., 1999), which would be the opposite of what was observed in our transcriptome analysis. In addition the signature analysis (Figure 3) which compares a variety of arrays with the one at hand found only weak resemblance to one array that tested for the effect of the VAM species *Gigaspora rosea* on *Arabidopsis* roots (Figure 3). Similar to mycorrhiza, bacteria colonizing *H. pilosella* roots would be encountered by *A. thaliana* roots and could have an impact on gene expression. In fact, different plant species are known to have specific root microflora (Hartmann et al., 2009). Well known examples of bacteria impacting on plant gene activity are *Pseudomonas fluorescens* inducing systemic resistance to pathogens (Pieterse et al., 1996, 1998; Léon-Kloosterziel et al., 2005) and *Bacillus subtilis* (Rudrappa et al., 2008) and *Paenibacillus alvei* (Tjamos et al., 2005) being involved in the induction of PR-proteins in *Arabidopsis*.

The second explanation is non-exclusive with the one above and is the perception of the neighbor root itself and/or its exudates. The possibility that exudates cause the pronounced transcriptomic effect and therefore play an essential role during neighbor perception is supported by results from the related field of kin recognition research. A study by Biedrzycki et al. (2010) demonstrated exudates from strangers and siblings to cause different root growth in *A. thaliana*. A subsequent transcriptome study found considerable impact of kin vs. stranger exudates on gene activity (Biedrzycki et al., 2011) and, even more interestingly, also found hints for the involvement of PR-genes in this process. Biedrzycki et al. (2011) found three genes with roles in pathogen defence among their 20 genes most induced by foreign exudates (*PDF1.3*, *PDF1.2b*, *CA1*). While these three genes were not significantly induced in our data set, some other 13 PR-coding genes were, and so was the entire functional category “PR proteins” (Figure 4). PR-proteins have different antimicrobial functions (antifungal, anti-Oomycete, chitinases, (1→3)-β-D-glucanases and others) but no clarified functions in plant-plant interaction. Their induction is the outcome of diverse and partly interconnected signal transduction pathways that involve different receptor proteins, plant hormones and transcription factors (Hammond-Kosack and Jones, 2000; Sels et al., 2008). Apparently, the induction of PR-proteins can thus be seen as a common outcome of pathogen perception and plant neighbor perception. This similarity, and especially the similarity between this data set and *Phytophthora*-affected transcriptome (Figures 3, 4), points to common features during the perception of microbes and neighboring plants.

Irrespective of whether the neighbor is perceived directly (root itself) or indirectly (associated microorganisms), the outcome for the *Arabidopsis* root is a reaction to the plant neighbor involving PR-proteins.

What cue elicits this reaction cannot be answered here. One might speculate about a possible role of oligogalacturonide (OGA) fragments from decomposition of neighbor roots' mucigels (Reymond et al., 1995; Ridley et al., 2001), because OGAs are known to bind to receptors located in the plasma membrane and to elicit plant defense responses including the induction of PR-proteins (Ridley et al., 2001; Aziz et al., 2007). Also, depending on concentration and species, they can both decrease or increase root growth (Bellincampi et al., 1993; Hernández Mata et al., 2006; Camejo et al., 2011). Considering the speculative nature of this, the exact molecular mechanisms of neighbor perception remain to be clarified.

### Genes responding to root interaction

Belowground interactions involve a lot of complex processes, most of which are hard to study. It would greatly facilitate research in root interaction to have good knowledge of genes that respond to interaction with neighbor roots. Data bases today hold no functional categories like plant-plant interaction, even though plant-plant encounters are ecologically highly relevant for plant fitness. Data presented by this paper show that root interaction affects a multitude of genes (6.4% in this case). The comparison of our data to the microarray data of Masclaux et al. (2012) (Figure 7; Tables 3, 4) basically represents a comparison of reactions in roots challenged by intraspecific competition on the one hand vs. interspecific interaction with *H. pilosella* on the other hand. This comparison also involves differences in the intensity of competition [strong in the setup by Masclaux et al. (2012), weak in our setup] and likely also in nutrient depletion [presumably strong in the setup by Masclaux et al. (2012), presumably weak in our setup]. In spite of these discrepancies, it was found that in induced genes there were more commonalities than would be expected just from proportionality. This makes sense biologically, because it appears that to some extent the perception of neighbors involves the same set of genes irrespective of whether the neighbor is of the same or of a different species. The transcripts concordantly regulated between the two setups might be considered a core group regulated in response to a broad range of neighbors. This core group comprises two signaling receptor kinases, genes involved in the regulation of transcription (incl. an induced myb-like transcription factor), and two pathogenesis-related proteins (namely the *RECEPTOR LIKE PROTEIN 54* and a Toll-Interleukin-Resistance domain family protein) (Tables 3, 4). Though this data can merely base on two studies, it is noteworthy that genes from these groups have a principal potential to perceive and transduce stimuli (by the signaling receptor kinases) and to regulate reactions (by the transcription factor) that include the induction of the named pathogenesis-related proteins.

At the same time, there are also large differences between the two data sets. While to a large extent these are likely due to the mentioned discrepancies in competition intensity and resource depletion between setups, it is also evident that some of the differences do potentially result from neighbor identities and reactions thereupon. A picture emerging from this is that in general the perception of neighbor roots involves a multitude of genes including numerous genes coding for pathogenesis-related proteins. A relatively small common core of genes is regulated during the very general perception of a neighbor, irrespective of its identity [e.g., the *RECEPTOR LIKE PROTEIN 54* (At5g40170)], while for the larger part of genes regulation depends on the specific identity of the neighbor.

To date, transcriptome data sets on interspecific root interaction are very rare, and comparisons are hampered by methodological differences. In a study on *Centaurea maculata* growing with either a strong or a weak competitor Broz et al. (2008) found 43 genes with significantly regulated transcripts. Among these, 26 genes were induced in the presence of a weak competitor, which is a situation remotely resembling our set-up. Just one of these genes was also significantly regulated in our data: the adenine nucleotide translocator, COR13 (At3g08580) was significantly repressed in both studies. Clearly, more studies with different species combinations are needed to identify genes that are typically involved in neighbor perception. A future challenge will be to establish sound knowledge on genes that respond to plant-plant interactions and—equally important—which genes respond differentially depending on the type of interaction, e.g., from competitive to facilitative, with distant to close relatives, under stressful to benign conditions. Further studies will also need to prove the robustness of these findings under different environmental conditions, e.g., different soil types. On the one hand it is clear that the perception of neighbors *per se* is a very common phenomenon, as reactions in root distribution caused by neighbor plants have been found by different studies on soil substrates as diverse as sand (Bartelheimer et al., 2006; Mommer et al., 2010), sand/topsoil mixture (Cahill et al., 2010), sand/loam/potting soil mixture (Mommer et al., 2010), agricultural soils like anthrosol (Li et al., 2006), and even artificial substrates like gel growth medium (Fang et al., 2013). However, the details and generality of our main finding (the similarity between transcriptional responses to plant neighbors and responses to pathogens) remain to be corroborated by further studies. The comparison of sterile to non-sterile conditions provides yet another approach to further elucidate the role of microorganisms in this context.

## Conclusions

For the inventory of its biotic environment a plant needs detection mechanisms to allow optimized morphological and physiological responses. The belowground presence of a neighbor largely impacts on root distribution and gene transcription. Transcriptome analyses in roots reveal pronounced similarities between responses to plant neighbors and responses to pathogens. Transcriptome comparisons between setups with intra- and interspecific interaction corroborate this finding and bring forward a core of consistently involved genes. This hints to conserved mechanisms and conserved responses to a broad range of biotic taxa encountered in the rhizosphere. In an ecological context it is revealed that a root may detect a neighbor directly or indirectly (by associated microfloras) without or before detecting resource depletion, which can save valuable time for the plant to prepare for potential interactions. Future studies need to explore transcriptional differences brought about by different neighbors and in different species.

### Conflict of interest statement

The authors declare that the research was conducted in the absence of any commercial or financial relationships that could be construed as a potential conflict of interest.
